# Supplementary material for: Generalized laws of thermodynamics in the presence of correlations
Source: Nat Commun. 2017 Dec 19;8:2180. doi: 10.1038/s41467-017-02370-x (PMC5736648; doi:10.1038/s41467-017-02370-x)
Supplement: Supplementary file 1 — Supplementary Information [file 41467_2017_2370_MOESM1_ESM.pdf]

SUPPLEMENTARY NOTE 1: DEFINITIONS OF HEAT

In the main text, the heat dissipated in a process involving a system and a bath B has been defined as $\Delta Q = kT\Delta\mathcal{S}_{\rm B}$, such as the common description: "flow of energy to a bath some way other than through work" suggests. Note, however, that this is not the most extended definition of heat that one finds in many works, e.g., [1, 2], where heat is defined as the change in the internal energy of the bath, i. e.

$$\Delta\tilde{Q} := -\Delta E_{\rm B}\,, \tag{1}$$

and no different types of energy are distinguished in this increase of energy. In this section, we compare these two definitions and argue why the approach taken here, though less extended, seems the most appropriate.

The ambiguity in defining heat comes from the different ways in which the change in the internal energy of the system $E_{\rm S}$ can be decomposed. More explicitly, let us consider a unitary process $U_{\rm SB}$ acting on a system-bath state $\rho_{\rm SB}$ with $\rho_{\rm B} = {\rm Tr}_{\rm S}\rho_{\rm SB} = \tau_{\rm B} \propto e^{-H_{\rm B}/kT}$ and global Hamiltonian $H = H_{\rm S}\otimes\mathbb{I} + \mathbb{I}\otimes H_{\rm B}$. The change in the total internal energy $\Delta E_{\rm SB}$ is the sum of system and bath internal energies $\Delta E_{\rm SB} = \Delta E_{\rm S} + \Delta E_{\rm B}$, or equivalently

$$\Delta E_{\rm S} = \Delta E_{\rm SB} - \Delta E_{\rm B}\,. \tag{2}$$

Many text-books identify in this decomposition $\Delta W := -\Delta E_{\rm SB}$ as work and $\Delta\tilde{Q} = -\Delta E_{\rm B}$ as heat. Nevertheless, note that it also assigns to heat increases of the internal energy that are not irreversibly lost and can be recovered when having a bath at our disposal.

To highlight the incompleteness of the above definition, let us consider a reversible process $U_{\rm SB} = \mathbb{I}\otimes U_{\rm B}$ that acts trivially on the system. Then, even though the state of the system is untouched in such a process, the amount of heat dissipated is $\Delta\tilde{Q} = -\Delta E_{\rm B} = {\rm Tr}\,[H_{\rm B}(\rho_{\rm B} - U_B\rho_{\rm B}U_{\rm B}^\dagger)]$.

In order to avoid this kind of paradoxes and in the spirit of the definition given above, we subtract from $\Delta E_{\rm B}$ its component of energy that can still be extracted (accessed). Then for a transformation $\rho_{\rm B}\to\rho'_{\rm B}$, the heat transferred is given as

$$\begin{aligned}\Delta Q &= -(\Delta E_{\rm B} - \Delta F_{\rm B}),\\ &= -kT\,\Delta\mathcal{S}_{\rm B},\end{aligned} \tag{3}$$

where $\Delta F_{\rm B} = F(\rho'_{\rm B}) - F(\rho_{\rm B})$ is the work stored on the bath and can be extracted. Here, $F(\rho_X) = E_X - kT\,\mathcal{S}(\rho_X)$ is the Helmholtz free energy, E_X is the internal energy and $\Delta\mathcal{S}_{\rm B} = \mathcal{S}(\rho'_{\rm B}) - \mathcal{S}(\rho_{\rm B})$ is the change in the bath's von Neumann entropy, $\mathcal{S}(\rho_{\rm B}) = -{\rm Tr}\,[\rho_{\rm B}\log_2\rho_{\rm B}]$. Throughout this work, we consider $\log_2$ as the unit of entropy.

Let us remark that in practical situations, in the limit of large baths, both definitions coincide. To see it, take Supplementary Eq. (3) and note that both definitions only differ in the free energy difference term, which together with the fact that the free energy is minimized by the thermal state, implies that the difference is very small when the bath is slightly perturbed. However, when studying thermodynamics at the quantum regime with small machines approaching the nanoscale such conceptual differences are crucial to extend, for instance, the domain of standard thermodynamics to situations where the correlations become relevant.

Note finally that both definitions express a path dependent quantity of the system like heat in terms of a difference of state functions of the bath. The path dependence character comes from the fact that there are several processes that leave the system in the same state but the bath in a different one. This connects with Clausius inequality, which is usually stated as

$$\oint \frac{{\rm d}Q}{T} \leqslant 0 \tag{4}$$

where the integral is taken over a cyclic path and the equality is only saturated by quasiestatic processes. In our framework and for the case of defining heat by means of information (entropy), the Clausius inequality is a consequence of the positivity of the mutual information. That is, by assuming global entropy preservation we have

$$\Delta\mathcal{S}_{\rm S} = -\Delta\mathcal{S}_{\rm B} + \Delta\mathcal{I}({\rm S}:{\rm B}) = \frac{\Delta Q}{T} + \Delta\mathcal{I}({\rm S}:{\rm B}) \tag{5}$$

where $\mathcal{I}({\rm S}:{\rm B}) = \mathcal{S}_{\rm S} + \mathcal{S}_{\rm B} - \mathcal{S}_{\rm SB}$ is the mutual information. For an initially uncorrelated system-bath, the mutual information can only increase $\Delta\mathcal{I}({\rm S}:{\rm B}) \geq 0$, and

$$\frac{\Delta Q}{T} \leqslant \Delta\mathcal{S}_{\rm S}\,. \tag{6}$$

For the definition of heat as an increase of the internal energy, we have

$$\Delta\tilde{Q} = \Delta Q - \Delta F_{\rm B} \leqslant \Delta Q\,, \tag{7}$$

where we have used Supplementary Eq. (3) and the positivity of the free energy change. In sum, for the case of initially uncorrelated states, we recover the Clausius inequality,

$$\Delta\tilde{Q} \leqslant \Delta Q \leqslant T\Delta S\,. \tag{8}$$

The deficit for the first inequality to be saturated is $\Delta F_{\rm B}$, that is, the energy that can still extracted from the bath. If one has a limited access to the bath, an apparent relaxation process will follow and the bath will thermalize keeping its energy constant. This will imply an entropy increase of the bath $\Delta F_{\rm B}/T$ which will make $\Delta\tilde{Q}$ and ΔQ coincide.

The deficit to saturate the second inequality in Supplementary Eq. (8) is $\Delta\mathcal{I}({\rm S}:{\rm B})$, that is, the amount of enabled correlations during the process. One of the main ideas of this work is to show that these correlations capture a free energy that can be extracted.

SUPPLEMENTARY NOTE 2: SET OF OPERATIONS

The set of operations that we consider in this manuscript is the so called entropy preserving operations. Given a system initially in a state ρ, the set of entropy preserving operations

are all the operations that change arbitrarily the state but keep its entropy constant

$$\rho \to \sigma \quad : \quad \mathcal{S}(\rho) = \mathcal{S}(\sigma)\,, \tag{9}$$

where $\mathcal{S}(\rho) := -\mathrm{Tr}\,(\rho \log \rho)$ is the Von Neumann entropy. It is important to note that an operation that acting on ρ produces a state with the same entropy does not mean that will also preserve entropy when acting on other states. In other words, such entropy preserving operations are in general not linear, since they have to be constraint to some input state. In fact, in [3], it is shown that a quantum channel $\Lambda(\cdot)$ that preserves entropy and respects linearity, i. e. $\Lambda(p\rho_1 + (1-p)\rho_2) = p\Lambda(\rho_1) + (1-p)\Lambda(\rho_2)$, has to be necessarily unitary.

One could think then that the extension of the unitaries to a set of entropy preserving operations is rather unphysical since they are not linear. However, they can be microscopically described by global unitaries in the limit of many copies [4]. That is, given any two states ρ and σ with equal entropies $S(\rho) = S(\sigma)$, then there exists a unitary U and an additional system of $O(\sqrt{n \log n})$ ancillary qubits such that

$$\lim_{n\to\infty} \|\mathrm{Tr}_{\,\mathrm{anc}}\left(U\rho^{\otimes n} \otimes \eta U^\dagger\right) - \sigma^{\otimes n}\| = 0\,, \tag{10}$$

where $\|\cdot\|$ is the one-norm and the partial trace is performed on the ancillary qubits. The reverse statement is also true, i. e. if two states can be related as in Supplementary Eq. (10) then they have equal entropies. This is proven in Theorem 4 of Ref. [4].

Sometimes it can be interesting to restrict entropy preserving operations to also be energy preserving. The set of energy and entropy preserving channels can also be described as a global energy preserving unitary in the many copy limit. More explicitly, in Theorem 1 of Ref. [4], it is proven that two states ρ and σ having equal entropies and energies ($S(\rho) = S(\sigma)$ and $E(\rho) = E(\sigma)$) is equivalent to the existence of some U and an additional system A with $O(\sqrt{n \log n})$ ancillary qubits with Hamiltonian $\|H_{\mathrm{A}}\| \leqslant O(n^{2/3})$ in some state η for which Supplementary Eq. (10) is fulfilled. Note that the amount of energy and entropy of the ancillary system per copy vanishes in the large n limit.

In sum, considering the set of entropy preserving operations means implicitly taking the limit of many copies and global unitaries. In addition, as that the set of entropy preserving operations contains the set of unitaries, any constraint that appears as a consequence of entropy preservation will be also respected by individual quantum systems.

The Hamiltonians of the system and the bath are the same before and after the transformation $\Lambda(\cdot)$. This can be done without loss of generality since, when this is not the case and the final Hamiltonian is different from the initial one, the two situations are related by a simple quench (instantaneous change of the Hamiltonian). More explicitly, let us consider a process (a) with equal initial and final Hamiltonian, and an identical process (b) with different ,

$$\text{(a)} \qquad (H, \rho_{\mathrm{i}}) \;\to\; (H, \rho_{\mathrm{f}}) \tag{11}$$

$$\text{(b)} \qquad (H, \rho_{\mathrm{i}}) \;\to\; (H', \rho_{\mathrm{f}}) \tag{12}$$

where $\rho_{\mathrm{i/f}}$ is the inital/final state, H the inital Hamiltonian and H' the final Hamiltonian of the process with different Hamiltonians. Then, it is trivial to relate the work and heat involved in both processes

$$W' = W + \mathrm{Tr}\,((H - H')\rho_{\mathrm{f}}) \tag{13}$$

$$Q' = Q\,, \tag{14}$$

where W' and Q' are the work and heat associated to the process (b) and we have only used that the process (b) is the composition of the process (a) followed by a quantum quench.

Let us finally point out that initially and finally the Hamiltonians of system and bath are not interacting, or in other words, the system is decoupled from the bath

$$H = H_{\mathrm{S}} \otimes \mathbb{I} + \mathbb{I} \otimes H_{\mathrm{B}}\,, \tag{15}$$

with $H_{S/B}$ the Hamiltonian of the system/bath. This is a necessary condition to be able to consider system and bath as independent systems each with a well defined notion of energy. Otherwise, assigning an energy to the system and to the bath would not be possible beyond the weak coupling limit. Note that the system and the bath interact (arbitrarily strongly) during the process, in which for instance a non-product unitary could be performed.

SUPPLEMENTARY NOTE 3: THE LANDAUER PRINCIPLE

The information theory and statistical mechanics have long-standing and intricate relation. In particular, to exorcise Maxwell's demon in the context of statistical thermodynamics, Landauer first indicated that information is physical and any manipulation of that has thermodynamic cost. As put forward by Bennett [5], the Landauer information erasure principle (LEP) implies that "any logically irreversible manipulation of information, such as the erasure of a bit or the merging of two computation paths, must be accompanied by a corresponding entropy increase in non-information-bearing degrees of freedom of the information-processing apparatus or its environment."

Following the definition of heat, it indicates that, in such processes, entropy increase in non-information-bearing degrees of freedom of a bath is essentially associated with flow of heat to the bath. The major contribution of this work is to exclusively quantify heat in terms of flow of information, instead of counting it with the flow of non-extractable energy, the work. To establish this we start with the case of information erasure of a memory. Consider a physical process where an event, denoted with i, happens with the probability p_i. Then storing (classical) information memorizing the process means constructing a d-dimensional system (a memory-dit) in a state $\rho_{\mathrm{S}} = \sum_i p_i |i\rangle\langle i|$, where $\{|i\rangle\}$ are the orthonormal basis correspond to the event i. In other words, memorizing the physical process is nothing but constructing a memory state $\rho_{\mathrm{S}} = \sum_i p_i |i\rangle\langle i|$ from a memoryless state $|i\rangle\langle i|$ where i could assume any values $1 \leqslant i \leqslant d$. On the contrary, process of erasing requires the transformation of a memory state

$\rho_S = \sum_i p_i |i\rangle\langle i|$ to a memoryless state $|i\rangle\langle i|$ for any i. Landauer's erasure principle (LEP) implies that erasing information, a process involving a global evolution of the memory-dit system and its environment, is inevitably associated with an increase in entropy in the environment.

In establishing the connection between information erasing and heat dissipation, we make two assumptions to start with. First, the memory-system (S) and bath (B) are both described by the Hilbert space $\mathcal{H}_S \otimes \mathcal{H}_B$. Secondly, the erasing process involves entropy preserving operation Λ^{SB}, i.e., $\rho'_{SB} = \Lambda^{SB}(\rho_{SB})$. The latter assumption is most natural and important, as it preserves information content in the joint memory-environment system. Without loss of generality, one can further assume that the system and bath Hamiltonians remain unchanged throughout the erasing process, to ease the derivations.

Now we consider the simplest information erasing scenario, which leads to LEP in its traditional form. In this scenario, a system ρ_S is brought in contact with a bath ρ_B and the system is transformed to a information erased state, say $|0\rangle\langle 0|_S$, by performing a global entropy-preserving operation Λ^{SB}, i.e.,

$$\rho_S \otimes \rho_B \xrightarrow{\Lambda^{SB}} |0\rangle\langle 0|_S \otimes \rho'_B, \tag{16}$$

where initial and final joint system-bath states are uncorrelated. The joint operation guarantees that the decrease in system's entropy is exactly equal to the increase in bath entropy and heat dissipated to the bath is $\Delta Q = -kT\,\Delta\mathcal{S}_B$. It clearly indicates that an erasure process is expected to heat up the bath. This in turn also says that $\Delta Q = kT\,\Delta\mathcal{S}_S$, where $\Delta\mathcal{S}_S = \mathcal{S}(\rho'_S) - \mathcal{S}(\rho_S)$. In the case where the d-dimensional system memorizes maximum information, or in other words it is maximally mixed and contains $\log_2 d$ bits of information, the process dissipates an amount $kT\log_2 d$ of heat to completely erase the information. In other words, to erase one bit of information system requires the dissipation of kT of heat and we denote it as one heat-bit or ℓ-bit (in honour of Landauer).

In the case where the final state may be correlated, the dissipated heat in general is lower bounded by the entropy reduction in the system, i.e.,

$$|\Delta Q| \geqslant kT\,|\Delta\mathcal{S}_S|. \tag{17}$$

This is what is generally known as the Landauer's erasure principle (LEP), in terms of heat.

The above formulation of LEP crucially relies on the fact that any change in system entropy leads to a larger change in the bath entropy, which is also traditionally known as the second law for the change in the information, i.e.,

$$\Delta\mathcal{S}_B \geqslant -\Delta\mathcal{S}_S. \tag{18}$$

However, it is limited by the assumptions made above and can be violated with initial correlations. Consider the examples in section of the Supplementary Information. In both the examples, $\Delta\mathcal{S}_B \ngeq -\Delta\mathcal{S}_S$. Therefore, one has to replace it with generalized informational second law.

SUPPLEMENTARY NOTE 4: VIOLATIONS OF LAWS OF THERMODYNAMICS

In order to highlight how the laws of thermodynamics break down in the presence of correlations, let us discuss the following two examples. In the first, the system S is purely classically correlated with the bath B at temperature T, while in the other they are jointly in a pure state and share quantum entanglement. In both the examples the Hamiltonians of the system and bath (H_S and H_B) remain unchanged throughout the processes.

Example 1 – Classical correlations.

$$\rho_{SB} = \sum_i p_i |i\rangle\langle i|_S \otimes |i\rangle\langle i|_B \xrightarrow{U^c_{SB}} \rho'_{SB} = |\phi\rangle\langle\phi|_S \otimes \sum_i p_i |i\rangle\langle i|_B,$$

Example 2 – Entanglement.

$$|\Psi\rangle_{SB} = \sum_i \sqrt{p_i}\,|i\rangle_S|i\rangle_B \xrightarrow{U^e_{SB}} |\Psi\rangle'_{SB} = |\phi\rangle_S \otimes |\phi\rangle_B,$$

where in both examples $|\phi\rangle_X = \sum_i \sqrt{p_i}\,|i\rangle_X$ with $X \in \{S, B\}$ and $1 > p_i \geq 0$ for all i. Note that the unitaries, U^c_{SB} and U^e_{SB}, leave the local energies of system and bath unchanged, and U^c_{SB} does not change the bath state.

A. Violations of first law

In Example 1, the Helmholtz free energy of the system increases $F(|\phi\rangle_S) > F(\rho_S)$ and therefore a work $-\Delta W_S = \Delta F_S > 0$ is performed on the system. To assure the energy conservation of the system, an equal amount of heat is required to be transferred to the bath. Surprisingly, however, no heat is transferred to the bath as it remains unchanged. Thus $\Delta E_S \neq -\Delta W_S + \Delta Q$, i.e. the energy conservation is violated and so the first law.

A further violation can also be seen in Example 2 involving system-bath quantum entanglement. In this case, a non-zero work $-\Delta W_S = \Delta F_S > 0$ has been performed on the system, and a heat flow to the bath is expected. In contrast, there is a negative heat flow to the bath! Therefore, it violates the first law, i.e. $\Delta E_S \neq -\Delta W_S + \Delta Q$.

B. Violations of second law and anomalous heat flows

We now show how correlation could result in a violation of the Kelvin-Planck statement of the second law, which states: No process is possible whose sole result is the absorption of heat from a reservoir and the conversion of this heat into work. In Example 1, no change in the local bath state indicates that there is no transfer of heat. However, the change in the Helmholtz free energy of the local system is $-\Delta W_S = \Delta F_S > 0$. Thus, a non-zero amount of work is performed on the system without even absorbing heat from the bath ($\Delta Q = 0$).

The situation becomes more striking in Example 2, with initial system-bath entanglement. In this case, $-\Delta W_S = \Delta F_S > 0$ amount of work is performed on the system. However, not only is there no heat flow from the bath to the system, but there is a negative heat flow to the bath! Thus, the second law is violated.

We next see how the presence of correlations can lead to anomalous heat flows and thereby a violation of the Clausius statement based second law. Such violations were known for the other definition of heat $\Delta Q = -\Delta E_B$ (see [2] and references therein). Here we show that such violations are also there with new heat definition $\Delta Q = -kT\Delta S_B$. Let $\rho_{AB} \in \mathcal{H}_A \otimes \mathcal{H}_B$ be an initial bipartite finite dimensional state whose marginals $\rho_A = \mathrm{Tr}_B \rho_{AB} = \frac{1}{Z_A}\exp[-\frac{H_A}{kT_A}]$ and $\rho_B = \frac{1}{Z_B}\exp[-\frac{H_B}{kT_B}]$ are thermal states at different temperatures T_A and T_B and with Hamiltonians H_A and H_B. In absence of initial correlations between the baths A and B, any energy preserving unitary will respect Clausius' statement of the second law. However, if initial correlations are present, this will not be necessarily the case.

Consider a state transformation $\rho'_{SB} = U_{AB}\rho_{AB}U^{\dagger}_{AB}$ where U_{AB} is a energy preserving unitary acting on ρ_{AB}. As the thermal state minimizes the free energy, the final reduced states ρ'_S and ρ'_B have increased their free energy,

$$\Delta E_A - kT_A\Delta S_A \geq 0 \tag{19}$$

$$\Delta E_B - kT_B\Delta S_B \geq 0\,, \tag{20}$$

where $T_{A/B}$ is the initial temperature of the baths, and $\Delta E_{A/B}$ and $\Delta S_{A/B}$ are the change in internal energy and entropy respectively.

By adding Supplementary Eqs. (19) and (20), and considering energy conservation, we get

$$T_A\Delta S_A + T_B\Delta S_B \leq 0\,. \tag{21}$$

Due to the conservation of total entropy, the change in mutual information is simply $\Delta\mathcal{I}(A:B) = \Delta S_A + \Delta S_B$, with $\mathcal{I}(A:B) = \mathcal{S}_A + \mathcal{S}_B - \mathcal{S}_{AB}$. This allows us to rewrite Supplementary Eq. (21) in terms of only the entropy change in A as

$$(T_A - T_B)\Delta S_A \leq -T_B\Delta\mathcal{I}(A:B)\,. \tag{22}$$

If the initial state $\rho_{AB} = \rho_A \otimes \rho_B$ is uncorrelated, then the change in mutual information is necessarily positive $\Delta\mathcal{I}(A:B) \geq 0$, and

$$k(T_A - T_B)\Delta S_A = -\Delta Q_A\frac{T_A - T_B}{T_A} \leq 0. \tag{23}$$

To see that this equation is precisely the Clausius statement, consider without loss of generality that A is the hot bath and $T_A - T_B > 0$. Then, Supplementary inequality (23) implies an entropy reduction of the hot bath $\Delta S_A \leq 0$ i. e. a heat flow from the hot bath to the cold one.

However, if the the system is initially correlated, the process can reduce the mutual information, $\Delta\mathcal{I}(A:B) < 0$, and Supplementary Eq. (22) allows a heat flow from the cold bath to the hot one.

C. Violations of zeroth law

The zeroth law establishes the notion of thermal equilibrium as an equivalence relation, in which temperature labels the different equivalent classes. To see that the presence of correlations also invalidates the zeroth law, we show that the transitive property of the equivalence relation is not fulfilled. Consider a bipartite system AC in an initial correlated state ρ_{AC}, like in Examples 1 and 2, and a third party B which is in a thermal state at the same temperature of the marginals ρ_A and ρ_C. Then, while the subsystems AB and BC are mutually in equilibrium, the subsystems AC are not, clearly violating transitivity. There are several ways to realize that the parties AC are not in equilibrium. One way is to see that any energy preserving unitary, except for the identity, decreases the amount of correlations between the parties, $\Delta\mathcal{I}(A:C) < 0$, which implies that the initial state is not stable. This can be shown from Supplementary Eq. (21) for the particular case of equal temperatures and the definition of mutual information. Another way is to see that the Helmholtz free energy follows $F(\rho_{AC}) > F(\rho_A \otimes \rho_C)$.

D. Violations of Landauer's erasure principle

Another thermodynamic principle that breaks down when correlations are present is Landauer's erasure principle. Landauer postulated that in order to erase one bit of information in the presence of a bath at temperature T, an amount of heat needed to be dissipated is $kT\log 2$. As noted in [1], when the system is classically correlated, there exists erasing process which does not increase entropy of the bath (see Example 1). The situation becomes more striking when the system shares quantum entanglement with the bath. This is the case of Example 2 with initial entanglement, where instead of increasing, an erasing process reduces the entropy of the bath and the bath is cooled down.

[1] David Reeb and Michael M Wolf, "An improved landauer principle with finite-size corrections," New Journal of Physics **16**, 103011 (2014).
[2] David Jennings and Terry Rudolph, "Entanglement and the thermodynamic arrow of time," Physical Review E **81**, 061130 (2010).
[3] Florian Hulpke, Uffe V. Poulsen, Anna Sanpera, Aditi Sen(De), Ujjwal Sen, and Maciej Lewenstein, "Unitarity as preservation of entropy and entanglement in quantum systems," Foundations of Physics **36**, 477–499 (2006).
[4] Carlo Sparaciari, Jonathan Oppenheim, and Tobias Fritz, "Resource theory for work and heat," Phys. Rev. A **96**, 052112 (2017).
[5] Charles H. Bennett, "The thermodynamics of computation—a review," International Journal of Theoretical Physics **21**, 905–940 (1982).
